# Investigation of social and cognitive predictors in non-transition ultra-high-risk’ individuals for psychosis using spiking neural networks

**DOI:** 10.1038/s41537-023-00335-2

**Published:** 2023-02-15

**Authors:** Zohreh Doborjeh, Maryam Doborjeh, Alexander Sumich, Balkaran Singh, Alexander Merkin, Sugam Budhraja, Wilson Goh, Edmund M-K Lai, Margaret Williams, Samuel Tan, Jimmy Lee, Nikola Kasabov

**Affiliations:** 1 Audiology Department, School of Population Health, Faculty of Medical and Health Sciences, The University of Auckland, Auckland, New Zealand; 2 Centre for Brain Research, The University of Auckland, Auckland, New Zealand; 3 School of Psychology, The University of Waikato, Hamilton, New Zealand; 4 Knowledge Engineering and Discovery Research Institute, School of Engineering, Computer and Mathematical Sciences, Auckland University of Technology, Auckland, 1010 New Zealand; 5 School of Psychology, Nottingham Trent University, Nottingham, UK; 6 Department of Psychology and Neuroscience, Auckland University of Technology, Auckland, New Zealand; 7 Institute for Stroke and Applied Neurosciences, Auckland University of Technology, Auckland, New Zealand; 8 Research Methods, Assessment & Science, Department of Psychology, University of Konstanz, Konstanz, Germany; 9 School of Biological Sciences, Nanyang Technological University, Singapore, Singapore; 10 Lee Kong Chian School of Medicine, Nanyang Technological University, Singapore, Singapore; 11 Center for Biomedical Informatics, Nanyang Technological University, Singapore, Singapore; 12 Department of Public Health and Psychosocial Studies, Auckland University of Technology, Auckland, New Zealand; 13 Institute of Mental Health & Lee Kong Chian School of Medicine, Nanyang Technological University, Singapore, Singapore; 14 Intelligent Systems Research Centre, Ulster University, Londonderry, UK; 15 Institute for Information and Communication Technologies (IICT), Bulgarian Academy of Sciences, Sofia, Bulgaria

**Keywords:** Biomarkers, Human behaviour, Psychosis

## Abstract

Finding predictors of social and cognitive impairment in non-transition Ultra-High-Risk individuals (UHR) is critical in prognosis and planning of potential personalised intervention strategies. Social and cognitive functioning observed in youth at UHR for psychosis may be protective against transition to clinically relevant illness. The current study used a computational method known as Spiking Neural Network (SNN) to identify the cognitive and social predictors of transitioning outcome. Participants (90 UHR, 81 Healthy Control (HC)) completed batteries of neuropsychological tests in the domains of verbal memory, working memory, processing speed, attention, executive function along with social skills-based performance at baseline and 4 × 6-month follow-up intervals. The UHR status was recorded as Remitters, Converters or Maintained. SNN were used to model interactions between variables across groups over time and classify UHR status. The performance of SNN was examined relative to other machine learning methods. Higher interaction between social and cognitive variables was seen for the Maintained, than Remitter subgroup. Findings identified the most important cognitive and social variables (particularly verbal memory, processing speed, attention, affect and interpersonal social functioning) that showed discriminative patterns in the SNN models of HC vs UHR subgroups, with accuracies up to 80%; outperforming other machine learning models (56–64% based on 18 months data). This finding is indicative of a promising direction for early detection of social and cognitive impairment in UHR individuals that may not anticipate transition to psychosis and implicate early initiated interventions to stem the impact of clinical symptoms of psychosis.

## Introduction

Over the last few decades, early identification and intervention of individuals at Ultra-High Risk (UHR) for psychosis has become a major translational research goal, in the hope of developing tailored methods to minimise the risk and impact of conversion to clinically relevant illness^1,2^. Whilst prediction of conversion to clinically relevant psychosis has been a main point of interest in the UHR populations, it is equally important to understand protective mechanisms present in UHR individuals who do not transition^3–5^. Social^6–9^ and cognitive^10–14^ functioning in UHR may have value in predicting illness progression. In particular, the covariation of social and cognitive measures across time might be useful in identifying and predicting outcome in UHR. To this end, the current study aims to apply novel computational neural network methods to longitudinal social and cognitive data in UHR to predict non-transition (i.e., remittance or maintenance of a nonclinical state).

A significant proportion of UHR individuals including those who remit from UHR conditions^15^, experience sustained declines in social functioning (i.e., real-life interpersonal behaviour, perceptual abilities, verbal and nonverbal communication)^6–8,16^. Deficits in social skills performance have been identified in schizophrenia. This included individuals at UHR for psychosis, individuals in early stages of psychosis, and individuals with chronic psychosis^17–19^. Studies of social functioning reported a higher degree of social skill impairment in UHR individuals who develop psychosis than in those who do not develop, although the lack of study power must be emphasised, which may indicate that a lack of social skills is a sign of susceptibility to psychosis^9,17^.

Cognition is another domain linked to poor performance in non-transition UHR individuals in follow-up studies^10,11^. This has been found within the specific neurocognitive domains of working memory, speed of processing, attention and executive functions^12–14^. Association between social and cognitive functioning factors to UHR individuals transitioning have been described cross-sectionally in the areas of theory of mind^8,20^, emotion recognition^21,22^ and attributional bias^8^.

To the best of our knowledge, no longitudinal studies have elucidated on social and cognitive interaction using advanced predictive tools in identifying and prognosis of non-transitioning UHR individual outcomes. This highlights the importance of longitudinal research on the predictive strength of symptom variables on social and cognitive data interaction that might have high translational potential in clinical psychiatric practice.

Advanced computational and machine learning methods have the potential to provide a method for development tools that can reliably predict individual health outcomes for multimodal and heterogeneous illnesses, such as schizophrenia^23,24^. Machine learning methods, such as pattern recognition, include computational algorithms that can learn from a large multivariate dataset to make accurate predictions concerning clinical outcome^25^. The potential of machine learning for prediction of individual outcomes in psychosis was illustrated in a study by de Nijs et al.^23^. The authors showed that patient-reportable baseline data (e.g., Global Assessment of Functioning (GAF) scores, psychotic symptoms, quality of life, antipsychotics use, psychosocial needs and depressive symptoms predicted global (accuracy 63.5–67.6%) and symptomatic (accuracy 62.2–64.7%) outcomes after 3-year and 6-year follow-ups. Worthington et al. (2022) applied a gradient boosting machine algorithm to clinical and demographic data to predict prodromal symptom remission in a UHR cohort. The classification accuracy was obtained at 60.0–72.0% with a sensitivity of 0.68% and specificity of 0.53%. The Worthington et al.’s study is notable as the first to examine interactive clinical and demographic predictors of symptomatic remission in people who do not progress to psychosis using an advanced data-driven techniques^26^.

The current study extends the use of data-driven model development to explore the use of a wide range of dynamic markers of social and cognitive variables to predict 2-year symptomatic outcomes and transition status. A subgroup of UHR’s individuals was included based on the clinical status at the final assessment (month 24) and compared them with the Healthy Control (HC) group. Examining the interaction and interrelations between social and cognitive variables over time required incorporating them into one unifying model^27^. To this end, the computational Spiking Neural Network (SNN) model was used for interaction and classification of social and cognitive data (using 2-year data for training and testing) and prediction (using 18 months of longitudinal data to test the model to predict the outcome at month 24). The results were also compared against other machine learning techniques that are suitable for time-series data for classifying and prognosis.

## Results

**Fig. 1:**
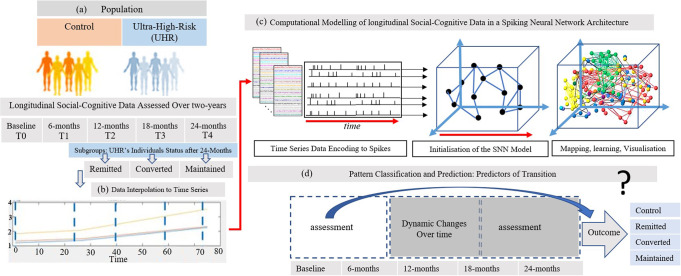
The diagram of the protocol of study includes population, data preprocessing, and the designed SNN-based methodology for visualisation, classification and prediction. **a** The longitudinal data that were measured from Ultrahigh risk individuals for psychosis and healthy control over a 2-year period; **b** interpolation of data points between the five measurements T0 (baseline), T1 (after 6 months), T2 (after 12 months), T3 (after 18 months) and T4 (after 24 months) to get the information-trend (time series); **c** Illustration of the designed methodology, containing encoding the changes in the time series into spike sequences and mapping them into a 3-Dimensional space of an SNN for learning, visualisation, and interaction; **d** computational modelling for classification and prediction of an individual transitioning outcome using dynamic two-years social and cognitive data.

The results from the designed SNN-based methodology (that is fully explained in the methods section), is depicted graphically in Fig. 1, and consists of the following steps:

1. Visualization of the interaction between social and cognitive variables.
2. UHR outcome classification and prediction.
3. Statistical analysis of the SNN models for assessing the model significance.

### Visualization and interaction of social and cognitive data

**Fig. 2:**
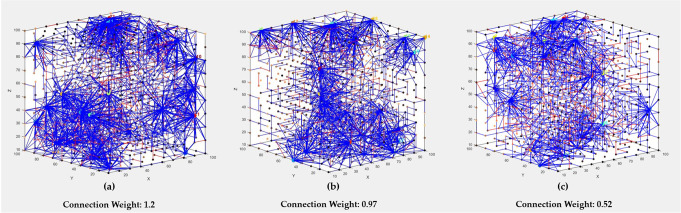
3D SNN connectivity models in the form of visualization on the longitudinal time series of social and cognitive data across groups (healthy control and UHR). Each model was mapped with a size of 1000 neurons (10 × 10 × 10 dimensions), which were then trained using the Spike Time Dependent Plasticity learning rule (STDP) to visualize 2-year social and cognitive data for **a** 81 HC samples; **b** 58 Remitter samples; and **c** 30 Maintained samples. For the variable mapping to SNN, the more correlated input variables (in terms of correlation in their spike trains) were mapped into nearby input neurons in SNN. The models were initialised using small-word connectivity with a radius set to 2 neuron distance, and then trained using STDP. The blue lines are positive connections (excitatory), and the red lines are negative connections (inhibitory) based on STDP learning. For each SNN model here, the average value of connection weight is reported as a metric below each model.

Figure 2a–c illustrate how the SNN models were trained on the longitudinal time-series of social and cognitive data across (a) HC group; (b) Remitter group and (c) Maintained group. The 23 social-cognitive variables (reported in Table 1) were mapped into a 3D SNN, where the spatial coordinates of the input neurons were defined based on the Graph Matching method^28^ (GM). The GM method positions highly correlated spike sequences of the 23 variables into topologically closer input neurons in the SNN. The intensities of the SNN connections vary across the variables for each group. To demonstrate the differences between the SNN models of HC and UHR subgroups, the average-value of the connection weights (*W*_*a*_) (Refers to the temporal relationship between two neurons as measured by the time of spike were extracted across every single social and cognitive variable.

**Table 1:** The 23 data variables along with the corresponding social and cognitive domains used in this study.

Variables numbers	Cognitive test: the Brief Assessment of Cognition in Schizophrenia (BACS) (9 variables)	Cognitive domains
1	List learning	Verbal memory
2	Digit sequencing	Working memory
3	Token motor task	Motor speed
4–7	Semantic fluency for animals, fruits, vegetables, and raw scores	Processing speed
8	Symbol coding	Attention and processing speed
9	Tower of London	Executive functions/reasoning and problem solving

	Social test: the High-Risk Social Challenge (HiSoC) (4 variables)	Social domain
10	Affect	Fluency of speech, engagement, social anxiety
11	Odd behavior and language	Facial affect, nonverbal affect, gaze, anergia
12	Social-interpersonal	Speech valence, appearance, clear communication
13	Averaged score	Total social score

	Cognitive test: snakes in the grass (SNK) (5 variables)	Cognitive domains
14–19	Reaction time score and accuracy score towards Target and distractor stimuli	Visual attention, inhibition, attentional bias, speed processing

	Cognitive test: perceptual closure (PC) (3 variables)	Cognitive domains
20–22	Filtered perceptual closure score and percentage of correctly identified items	Energizing or arousing, attention to the complete figure, ability to maintain a major set.

	Cognitive test: continuous performance task (CPT) (1 variable)	Cognitive domains
23	Averaged score	Sustained and selective attention

**Fig. 3:**
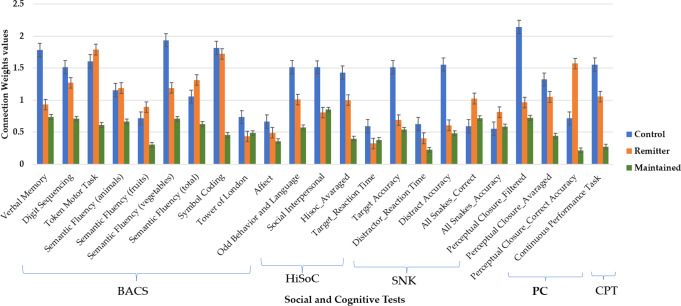
The average of connection weights within 23 clusters of neurons inside the SNN, each of which includes neurons that are directly connected to one of the 23 social-cognitive variables, across three groups: Control group (blue bar), Remitter group (orange bar), and Maintained group (green bar). The components of cognitive BACS test include verbal memory, digit sequencing, token motor task, semantic fluency, symbol coding, and tower of London; the components of social HiSoC test include affect, odd behaviour, and language, and social impersonal; the components of cognitive SNK test include target and distractor accuracy and reaction time; the components of cognitive PC include the filtered, averaged and accuracy of perceptual closure scores; and the cognitive CPT test include the total score.

The average-value of connection weights in the SNN models of HC and Remitter groups were greater (*W*_*ij*_: 1.2 and *W*_*ij*_: 0.97 respectively) than the Maintained group (*W*_*ij*_: 0.52). In addition to the average-value of all the connection wights in each SNN, we also reported a greater detail on the SNN connections in Fig. 3 that better demonstrates the differences between the models: (1) “HC vs Remitter” and (2) “HC vs Maintained”. Figure 3 shows the average-value of connection weights for each variable separately. This is calculated by taking average of connection weights among neurons which are directly connected to the SNN input variables (social and cognitive variables).

As shown in Fig. 3, the most prominent discriminative cognitive variables between HC and Remitters are verbal memory and semantic fluency (vegetables) from the Brief Assessment of Cognition in Schizophrenia (BACS) test; filtered score in Perceptual Closure (PC) test; and target and distractor accuracies scores in the Snakes in the grass (SNK) test. The discriminative social variables between HC and Remitters are odd behaviour, language, and social interpersonal from the High-Risk Social Challenge (HiSoC) test. These cognitive variables and the corresponding functions including verbal memory, processing speed, attention, and inhibition; and social functioning, including communication and fluency of speech can be suggested as potential predictive markers for identifying Remitters.

Figure 3 shows that the performance of HC and Maintained groups are different across verbal memory, digit sequencing, token motor task, semantic fluency, and symbol coding from the BACS test; target and distractor accuracies from (SNK) test; all the variables from the PC test and the total score for the Continuous Performance Task (CPT). The social skills performance also differs across HC and Maintained in affect, odd behaviour, language, and social interpersonal from the HiSoC test. These findings suggest that the cognitive and social functions, including verbal memory, working memory, motor speed, processing speed, attention, inhibition, nonverbal affect, communication and fluency of speech are the most discriminative and predictive markers for identifying the Maintained subgroup.

**Fig. 4:**
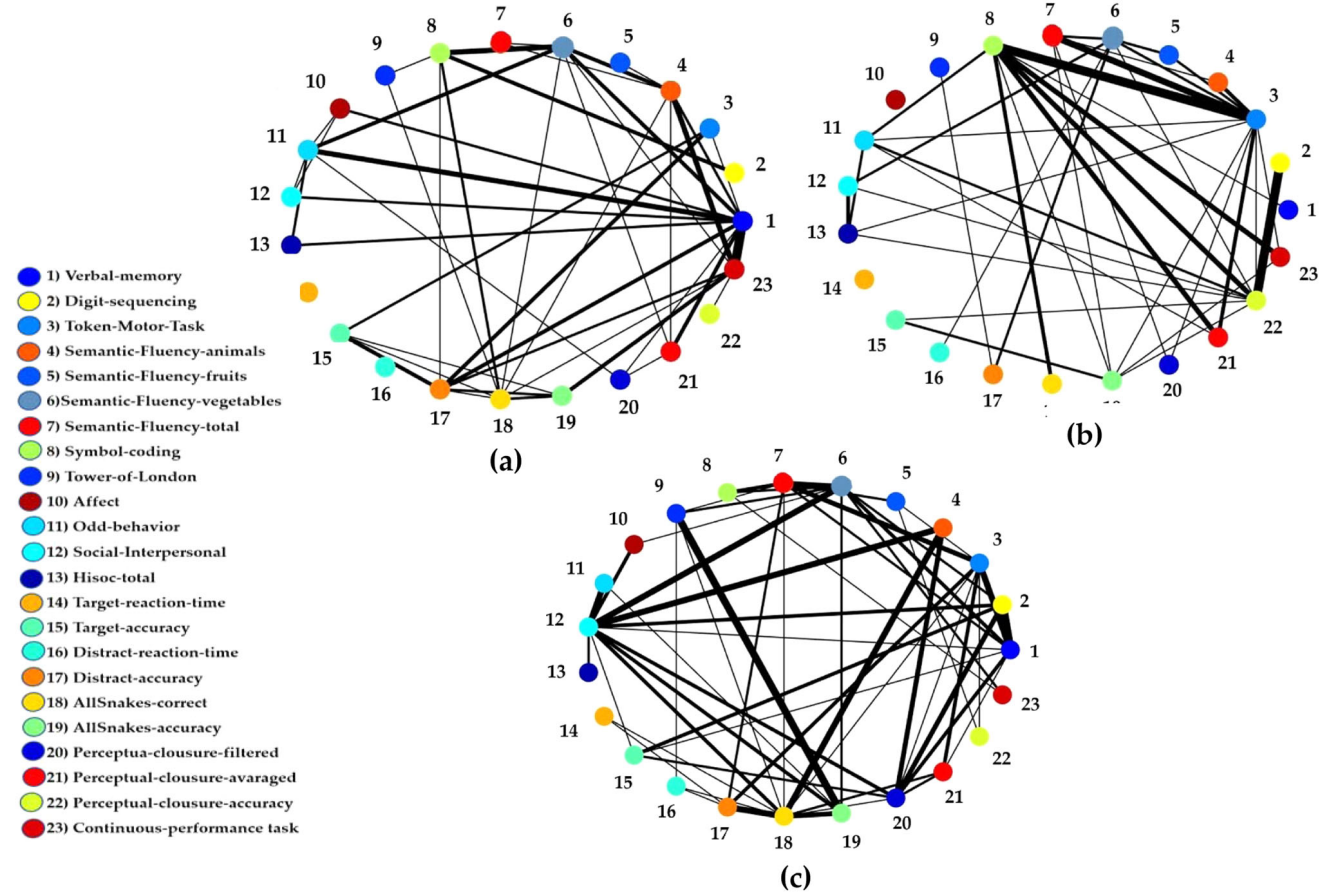
The amount of spike interaction during the Spike Time Dependent Plasticity (STDP) learning in the SNN between each two variables from the 23 social and cognitive variables through Feature interaction network (FIN) Model. **a** HC group; **b** Remitter group; and **c** Maintained group. Each node represents a cluster of neurons around one variable (connected directly inside the SNN). Each line represents how two clusters of neurons (representing variables) exchanged spikes during STDP learning. The thicker the line between two variables, the greater the level of spike. interaction between them.

The significance of the trained SNN models across HC and UHR subgroups were examined by calculating the number of spikes that were exchanged between the cognitive and social variables. This is demonstrated by Feature Interaction Network (FIN)^29^ is shown in Fig. 4. Here, every node represents a cluster of spiking neurons that are centred by one data variable. The neurons are clustered using a dynamic evolving clustering technique which is based on network theory^30–32^. The lines in FIN demonstrate spikes were exchanged between neurons of adjacent clusters during the Spike Time Dependent Plasticity (STDP) learning. The thicker lines suggest greater number of transmitted spikes, representing more temporal interaction and greater exchange of information between the variables. A stronger social and cognitive interaction in the Maintained group is shown in Fig. 4c in comparison with other groups (Fig. 4a, b). This finding can be used to study the causal interaction between variables over time and to predict important variables that may affect other variables to change in UHR individuals’ status.

### Classification and Prediction of UHR’s individuals’ outcomes

For identification of UHR individuals, we applied classification using SNN classifier of dynamic SNN (explained in the Method section). There is a total number of 169 samples across three classes (*n* = 81 HC; *n* = 58 Remitters; and *n* = 30 Maintained). The classification was performed based on 30 times of cross-validation through a random 50–50% split. The balance accuracy is reported in Table 2a. A predictive modelling experiment was also carried out to investigate how early the SNN can capture the discriminative patterns of changes in social and cognitive data between groups for the purpose of predicting the individual outcomes. The class label for each individual’s data is given in relation to the outcome diagnosed in 24-month. An SNN model was tested (using only the data collected during the first 18 months) to predict the output classes for the three groups of participants at the time of 24-month (label as HC, Remission and Maintained at 24-months).

**Table 2:** Classification and Prediction of Healthy Control and UHR’s Individuals’ Outcomes Using Spiking Neural Network.

(a) Classification accuracy outcome						
Group	Control	Remitted	Maintained	Total accuracy	Standard deviation	
Accuracy %	0.89	0.71	0.64	0.78	0.2	

(b) Prediction accuracy outcome						
Group	Control	Remitted	Maintained	Total accuracy		Standard deviation
Accuracy %	0.94	0.68	0.68	0.80		0.2

(a) Classification of 169 samples into three classes: HC (class 1, containing 81 samples), Remitter group (class 2, containing 58 samples, and maintained group (class 3, containing 30 samples. Class labels are extracted from T4 (at 24 month). For classification, the whole length of cognitive and social time series (T0-T4) was used in the training and testing sets. The results are the average of balance accuracies from 30 rounds of 2-fold cross-validation. (b) Prediction of three classes (HC, Remitted, and Maintained, labels are extracted from 24 month). For prediction, the length of cognitive time series in testing sets was 18 months (only T0-T3) to predict the outcome at month 24. This is to predict which individual is likely to remit or maintain the level of social- cognitive statues at time T4 (at 24-months of assessment) when the SNN model was only tested by the data from earlier time (18-monthss). The SNN parameters set as the following: Encoding threshold: 0.50; Firing threshold: 0.50; STDP learning rate: 0.01; Mod: 0.8; Drift: 0.005.

The prediction experiment was based on running a 2-fold cross-validation (random 50–50 split) 30 times, and the average of all 30 balance accuracies was calculated (reported in Table 2b). The 6-month ahead prediction of outcomes (HC, Remission and Maintained) is reported as 80.2% balance accuracy (Table 2b).

The classification and prediction were also conducted using other traditional methods, including k-nearest neighbours (KNN), random forest, and support-vector machine (SVM) through a random 50–50% split performing 30 times. Here, for each individual’s data sample (which is interpolated to time series), all the variables’ time points were concatenated into a single vector, which unlike the SNN model that can reactive temporal data, disregards the intrinsic temporal structure of the interpolated social and cognitive data. To perform a fair comparison, we also applied long short-term memory (LSTM) and Convolutional neural network (CNN) method that can learn from streaming timeseries (Table 3a, b).

**Table 3:** Classification and Prediction of UHR’s individuals’ outcomes Using Machine Learning Tools.

(a) Classifying between Remitter and Maintained						
Algorithm	Accuracy %	F1	MCC	AUROC	Sensitivity	Specificity
LSTM	0.593 Sd (0.07)	0.231 (0.17)	0.019 (0.16)	0.493 (0.08)	0.787 (0.16)	0.228 (0.19)
CNN	0.524 (0.12)	0.294 (0.18)	0.012 (0.12)	0.498 (0.06)	0.628 (0.34)	0.384 (0.35)
SVM	0.625 (0.07)	0.253 (0.14)	0.063 (0.17)	0.524 (0.07)	0.839 (0.09)	0.219 (0.31)
Random Forest	0.596 (0.09)	0.220 (0.16)	0.047 (0.21)	0.518 (0.07)	0.868 (0.10)	0.166 (0.31)

(b) Predicting between Remitter and Maintained (Prognosis) Baseline to 18 months data and labels at 24 months						
Algorithm	Accuracy	F1	MCC	AUROC	Sensitivity	Specificity
LSTM	0.569 (0.07)	0.175 (0.14)	−0.050 (0.12)	0.474 (0.08)	0.783 (0.17)	0.176 (0.18)
CNN	0.556 (0.09)	0.291 (0.15)	0.005 (0.12)	0.492 (0.07)	0.664 (0.24)	0.335 (0.25)
SVM	0.641 (0.07)	0.280 (0.17)	0.079 (0.20)	0.533 (0.08)	0.825 (0.10)	0.241 (0.16)
Random Forest	0.633 (0.08)	0.288 (0.13)	0.114 (0.19)	0.541 (0.07)	0.842 (0.12)	0.241 (0.13)

(a) Classification results of two classes (Remitter and Maintained) using machine learning tools including Random Forest, Support vector machine (SVM), K-nearest neighbour (KNN), and Long Short term memory (LSTM) and Convolutional neural network (CNN).

(b) The prediction task was also conducted using Random Forest, SVM, KNN, LSTM, and CNN. The *F* score, Matthew’s correlation coefficient (MCC), Area Under the Curve (AUROC) for measuring the performance of the machine learning tools are also reported.

### Statistical analysis

**Fig. 5:**
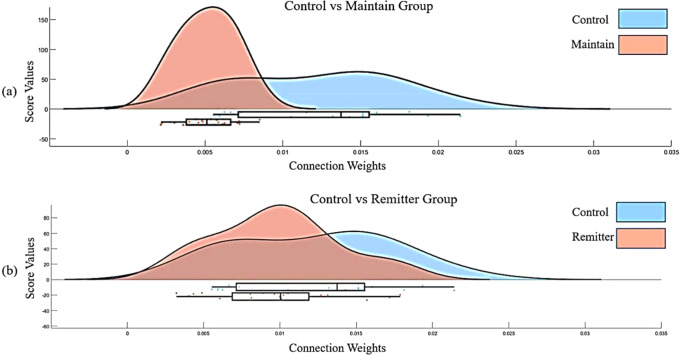
A raincloud plot of the connections weights as a function of group (healthy control and UHR) and social and cognitive performance. **a** Distribution of the connection weights comparison between healthy control and maintained groups. **b** Distribution of the connection weights comparison between HC and Remitter groups.

To evaluate the significance of the trained SNN models, a Multivariate Analysis of Variance (MANOVA) tested for differences across groups in connection weights, separately for the social and cognitive tests. Between groups variables included Sex (male, female) and Group (HC, Remitter, Maintained). Sub scores from each test were entered as separate dependent variables. Greenhouse-Geisser corrections were used to correct all violations of the assumption of sphericity. There was a significant main effect of *Group* in the PC test [*F* (3) = 3.1, *p* < 0.03, *ƞ*^2^ = 0.05]. There was a significant effect of *Sex* in PC sub-score (percentage of correctly identified items), with higher connection weights in men than women. There was a significant Group × Sex interaction effect for HiSoC Affect Dimension [*F* (2) = 3.07, *p* = 0.05, *ƞ*2 = 0.04] and HiSoC Social Interpersonal Dimension [*F* (2) = 3.21, *p* < 0.04, *ƞ*2 = 0.04]. Post-hoc tests showed higher scores for Affect in male Remitters compared to Maintained (*p* < 0.04). In women, on-the-other-hand, the Social Interpersonal Dimension was better in Remitters than Maintained (*p* < 0.01). Figure 5 shows a raincloud plot of the connections weights as a function of group and social and cognitive performance.

## Discussion

Using a rigorous computational SNN approach, we developed models to investigate the dynamic social and cognitive interactions and to predict 2-year outcomes of UHR’s individuals for psychosis. Findings identified the most important cognitive and social variables (particularly verbal memory, processing speed, attention, affect and interpersonal social functioning) that showed discriminative patterns in the SNN models of HC vs UHR subgroups. The explainable SNN model enabled us to have a better understanding of the interaction of data variables across groups over time (with higher social and cognitive variables interaction for UHR Maintained subgroup than Remitter subgroup). Finally, the accuracy of social and cognitive data classification was higher when using SNN models compared with traditional machine learning methods (e.g., SVM, LSTM, Random Forest and CNN).

### UHR state for psychosis and non-transition

Longitudinal modelling of social and cognitive performance revealed that the majority of individuals (64.5%) were remitted during the 24-month follow-up, while about 34% of the individuals maintained their UHR status and only 2% converted to clinically relevant psychosis. These results were consistent with more recent studies that reported that the rate of transition from an UHR status to clinically relevant psychosis has dropped dramatically from over 50% to 15% with follow-ups exceeding 1 year^5^.

### Dynamic social and cognitive changes and interactions

The results showed that participants at UHR for psychosis were differentiated from HC, and Remitters were differentiated from Maintained according to 24-months social and cognitive performance. The total connection weights value showed Control > Remitter > Maintained (Fig. 2a–c). In particular, greater connectivity weights were generated for the “PC” cognitive test in the HC group than in other UHR subgroups. Greater connection weights here reflect better performance. The “PC” task tests ability to complete what is incompletely presented in the visual field to achieve “good” or as complete a figure as possible by perception^33^. PC scores have previously been found lower in well-established (chronic) schizophrenia than the general population, and was interpreted as reflecting atypical attention, or inability to maintain a major set^33^.

In between group comparison, HC and Remitter groups differed slightly in the connectivity of symbol coding from BACS test and affect dimension from HiSoC test. This suggests cognitive and social functions in the Remitter group (attention, processing speed and affect) returned to a similar level as the HC after 24 months. Moreover, the connection weight values of some of the cognitive sub-variables increased for the Remitter group to some different degrees. For example, the connection weights for the PC test (correct answer accuracy), SNK (total score accuracy) and BACS (semantic fluency in fruits and animals and token motor task) were higher than in the HC at 24-months. These variables correspond to organisation, visual, motor speed and processing speed domains, respectively. These results were consistent with Kendler et al. (2016) study that reported an improved cognition in individuals at UHR with time^34^.

The difference between the HC group and the Maintained subgroup are across all the social and cognitive variables except SNK test performance. The connection weight values of SNK sub-variables increased slightly for the Maintained group in relative to HC group. These variables represent cognitive abilities related to visual attention and processing speed. An increase in variability of test performance over time suggest the possibility that the underlying cognitive architecture may have devolved in Maintained group during follow-up. Thus, measures of dedifferentiation of cognitive components may be one of the most powerful factors in later conversion in individuals at risk for psychosis^35^.

Through statistical analysis of the extracted connection weights, significantly higher connection weights were seen in the Remitters group for HiSoC social interpersonal dimension than in other groups at 24 months that reflect greater co-variation between social interpersonal score and other cognitive measures with time. There was also a significant effect of sex in HiSoC as the Affect dimension was protective in male Remitters and the Social Interpersonal dimension was protective in female Remitters. These findings are in alignment with McGlashan^36^ that found females had better premorbid social functioning than males. Vila-Rodriguez et al.^37^ expanded these studies and discovered that females performed better in social interpersonal skills than males. These findings suggest that social interpersonal functioning in females and affective functioning in males could be the most important social variable to differentiate the groups and sex and can be used for predicting progression.

Through FIN analysis in Fig. 4, for the Maintained group, a stronger exchange of information between social and cognitive variables was seen, suggesting that dynamic variations in social and cognitive performance interact differently with time in the different groups. This interaction was more between BACS and SNK from cognitive functioning and interpersonal and odd behaviour from social functioning. According to STDP rule in SNN model, connectivity with greater weights reflects stronger spike transmission between inputs. Thus, the STDP rule develops neural connectivity among the spatially distributed inputs in the SNN model that represents the spatiotemporal relationships between the social and cognitive data variables. Finding is Fig. 4 indicates the strength of intercorrelation between cognitive and social variables and reflect how particularly attention and processing speed deficit in Maintained group can affect the dysfunctionality of other aspects of social skills including communication, and fluency of speech.

### UHR’s classification and prediction

The classification of social and cognitive data over 2-years resulted in significantly higher accuracy across groups, compared to other machine learning methods (Table 2a). The prediction accuracy of dynamic social and cognitive data for Remitter and Maintained groups was 80.2% when only 18-months of data was used which suggested the existence of discriminative patterns between the groups at one year and half earlier (Table 2b). Compared to commonly applied predictive risk models for UHR which are based on modelling static variables, the current study is one of the newly advanced studies on developing a temporal-based risk prediction model using longitudinal social and cognitive data and SNN. With machine learning techniques, the spatiotemporal variables of social and cognitive data were converted into one vector of variables. As a result, temporal connections cannot be obtained. In comparison, the computational SNN models allow the integration of dynamic interactions between social and cognitive performance during various neuropsychological tasks performance.

### Limitations and future work

There are some limitations to consider. First, we only selected the common social and cognitive variables that were collected across all the five times of follow-ups; thus, we ended up using 23 social and cognitive variables. Second, the SNN models were trained and tested on a small set of data and the findings are valid for the defined scope. However, we demonstrated the potential of our model in UHR’s predictive marker detection and 6-months ahead prediction with a better balance accuracy compared to other machine learning tools. Therefore, the SNN model needs to be further tuned and calibrated using more data for the generalisation purpose. Further longitudinal studies are needed to look at a time period over the 24 months done in the current study with a larger cohort population. Future research might look at the non-transitioned individual differences in time, that is affected by other variables including ethnicity, and culture. Of the ethnic groups reported, schizophrenia is a culture-bound illness, that is, a difference in culture can influence how it manifests. The results of this paper will be further extended through developing a SNN model to learn from multimodal data including genetic, neuroimaging, clinical along with social and cognitive longitudinal data. We plan to conduct personalised profile modelling for every individual using the personalised modelling approach. Our future work also includes enhancing the interpolation phase by optimising the number artificial datapoints between original data measurements.

In conclusion, the research demonstrated the feasibility of a computational SNN approach for longitudinal outcome prediction, in the most difficult to prediction, a non-transition UHR population for psychosis, based on social and cognitive data interaction. Our findings support the importance of temporal changes (over two years) and the co-variation across certain social and cognitive variables in predicting outcomes. The SNN models resulted in a good balanced accuracy for 6-month ahead prediction of UHR outcomes (based on 18-month data) for individuals who are likely to remit and for those who are likely remain stable at 24-month period. Thus, the designed SNN architecture in this study can be suggested as a possible supportive tool for early detection of UHR individuals. The SNN approach has shown to be potential in modelling UHR data. This can benefit psychiatrists and other health professionals in identifying non-converted individuals and allow them to initiate early and appropriate targeted interventions.

## Method

### Participants and inclusion criteria

The original study conducted in Singapore included *n* = 384 HC individuals (mean age = 21.7 years and SD = 3.4) and *n* = 173 UHR individuals (mean age = 21.3 years and SD = 3.5). All participants were between the ages of 14 and 29.

#### Current inclusion criteria

Only individuals who had their data collected from all variables across all five time points T0-T4 were included in the current study. Thus, a total number of 171 individuals were selected for UHR group (*n* = 90) and HC group (*n* = 81); age mean =21.29 and SD = 3.70; 106 males and 65 females. UHR individuals were then categorised into three classes (Remitters, Converters, and Maintained), based on the clinical scores at 24-month follow-up (Fig. 1a). Remitters (*n* = 58) refers to those UHR participants who were UHR-positive at baseline but did not meet the UHR criteria at the 24-month. Converters (*n* = 2) refers to those participants who were UHR-positive at baseline and developed a first episode of psychosis within 24-months. Maintained (*n* = 30) refers to those participants who were UHR-positive at baseline and remained UHR-positive at 24-month period of follow-up. The Converter group was dropped from the current study due to low sample size. Hence, the final study dataset contained 169 individuals (HC, Remitters, Maintained).

### Assessment

Multimodal data (demographic, clinical, social, and cognitive) were collected every six months over two years: T0 = baseline assessment, T1 = 6-months, T2 = 12-months, T3 = 18-months and T4 = 24-months follow-ups. In total, twenty-three social and cognitive variables were identified (reported in Table 1) that were determined as common variables collected at baseline (T0) and at all follow-up assessments (T0 - T4).

#### Clinical assessments

The Comprehensive Assessment of At-Risk Mental States (CAARMS) was employed to determine the UHR’ clinical status^38^. The UHR subgroup was then categorised into three classes (Remitters and Maintained), based on the CAARMS scores at 24-month follow-up.

#### Social assessment

The performance-based social skills was measured by the High Risk Social Challenge Task (HiSoC)^39^. The HiSoC has been used in UHRs for psychosis studies as a standardized and performance-based social skills measure that has demonstrated high levels of validity and reliability. Social skills are measured in terms of the display of affect, odd behaviour, and language, and social-interpersonal when evaluating the task. A 5-point Likert scale is used to rate the 16 items in the task (with higher scores indicating better social skills).

#### Cognitive assessment

The cognitive assessments are based on four cognitive neuropsychological batteries, including (i) The Brief Assessment of Cognition in Schizophrenia (BACS)^40,41^ which comprises list learning, digit sequencing, token motor task, semantic fluency, Tower of London, and symbol coding tests; (ii) The Snakes in the grass (SNK)^42,43^ test of fear-relevant attention (accuracy and reaction time); (iii) The Continuous Performance Test (CPT)^44^;and iv) Perceptual Closure test (PC)^45^.

### Data analysis

#### Interpolation

To capture the 2-year trend in these 23 social-cognitive variables across groups, we applied an interpolation technique which is a common practice in clinical data generation^46,47^. There are examples of successfully applying interpolation techniques that improved the modelling of changes in limited measured datasets, including hippocampus-amygdala in schizophrenia^48^, heart rate measurement in atrial fibrillation^49^, and MRI in traumatic brain injury^50^ and MRI modelling in prediction of dementia^51^. Durrleman et al. shows estimated timepoints (values) generated by interpolation in a different range of longitudinal datasets inferred continuous shape trajectory from observations sparsely distributed in time^52^. One of the interpolation methods is linear interpolation that requires knowledge of two data points and assumes constant rate of change between them. This is a process of using known data values to estimate unknown data values. In the current study, a linear interpolation is used to generate the values between the limited number of data measurements (T0-T4) in 2 years. The interpolated data points do not alter the trend of original data and only depict the linear pattern of changes between the five follow-up measurements. In the current study, a linear interpolation is used to generate the values between the limited number of data measurements (T0-T4) in 2 years. The interpolated data points do not alter the trend of original data and only depict the linear pattern of changes between the five follow-up measurements. Interpolation technique allows to generate artificial data point per month, transforming the data from static domain to time series while preserving the original trend of changes across groups. This led to generate 245 times points (time series) for each variable associated with 24 months, plus 5 time-points from the original data collection as shown in Fig. 1b. The interpolation of all the social and cognitive variables to time series, shown in Supplementary Fig. 1, represents the trends in the time series and the gradual changes. This interpolation is conducted prior to modelling the data using machine learning models and SNNs (explained in the following section). For SNN models, the interpolated time series were then encoded into sequences of binary events, called spikes that capture significant upward and downward changes in the 2-year social-cognitive data.

#### Computational spiking neural network modelling

The SNN model has been developed as a connectionist network framework that both spatial and temporal components of data can be learnt and trained in one model. The SNN model has a 3-dimensional structure of artificial spiking neurons^28^ and a learning algorithm that learns from streaming data and adapts the connection weights, representing spatio-temporal relationship between variables^30^. Interpolated social and cognitive variables are used as input^30^ to the SNN model to learn the patterns of changes in longitudinal data in relation to the outcomes (HC and UHR subgroups).

The data modelling architecture possesses the following modules:

(i) Input module: a threshold-based encoding algorithm^53^ was applied to the interpolated timeseries of each variable and converted them into sequences of spikes, representing significant changes in each variable values that occurred over time. This results in a compact representation of events (spikes) and removing noise. The variables were spatially mapped into input neurons in the SNN to feed the model with the spike sequences. The 3D mapping of the SNN and the initialisation procedure are explained Supplementary material.
(ii) SNN training module: a twofold learning process is applied. First, the input spikes are passed into the SNN model for unsupervised learning using Spike Time dependent Plasticity (STDP)^54^ (Fig. 1c). Then, the trained SNN model is connected to an output layer for supervised learning to capture associations between the trained SNN model and the training sample class labels (HC and UHR subgroups). The supervised learning is based on a Dynamic Evolving SNN (deSNN), shown in (Fig. 1d)^55^. The detains of the STDP is explained in Supplementary material.
(iii) Classification and prediction module: the trained SNN model was tested using the social and cognitive data of those individuals who were excluded from the training process. The testing and training sets were defined, based on a 2-fold cross validation in which 50% of samples were selected randomly as the training set, while and other 50% hold-out samples were used as testing set. This process was repeated 30 times and the random selection approach assured sample permutation. For the classification task, the whole temporal length (T0-T4) of the social and cognitive variables were used for training and testing to detect the outcomes (in month 24). However, for the prediction task, only the first 18 months (T0-T3) of the data were used to test the model for performing a 6-month-ahead prediction of outcomes (at month 24). Since the dataset is imbalanced (Remitted *n* = 58 and Maintained *n* = 30), balanced accuracy is calculated as (Sensitivity + Specificity) / 2, reported in confusion Table 2., representing the number of correctly classified and miss-classified samples.
(iv) Visualisation, interaction and knowledge discovery module: in the trained SNN, the spatiotemporal connections between the social and cognitive variables were visualised and traditional statistics used to test for differences between groups. Differences between HC and UHRs subgroups in SNN models were also studied by computing spatio-temporal interactions between the social-cognitive variables, using a Feature Interaction Network (FIN).

Figure 1a–d presents the protocol of study, as well as the designed computational SNN-based methodology for data modelling, visualisation, interaction, classification and prediction. The details of the designed SNN computational models including mathematical formulas and algorithms are presented in the Supplementary Material, Section 1.1.

### Ethical approval

All aspects of the study were completed in alignment with appropriate regulations and guidelines of Nature journal publishing. Ethical approval was granted by the Singapore National Healthcare Group’s Domain Specific Review Board. Informed consent was obtained from all participants. For those under 21 years of age, the consent was obtained from a legal representative.

## Software availability

The software used for the implementation of the designed method can be found at http://www.kedri.aut.ac.nz/neucube/.

## Supplementary information

Investigation of Social and Cognitive Predictors in Non-Transition Ultra-High-Risk’ Individuals for Psychosis Using Spiking Neural Networks

## Data Availability

Dataset is not publicly available due to participant consent statement but could be available from the corresponding author upon reasonable request and with permission of NTU and IMH, Singapore, considering a data sharing agreement procedure.
