# Supplementary material for: Investigation of social and cognitive predictors in non-transition ultra-high-risk’ individuals for psychosis using spiking neural networks
Source: Schizophrenia (Heidelb). 2023 Feb 15;9(1):10. doi: 10.1038/s41537-023-00335-2 (PMC9931713; doi:10.1038/s41537-023-00335-2)
Supplement: Supplementary file 1 — Investigation of Social and Cognitive Predictors in Non-Transition Ultra-High-Risk’ Individuals for Psychosis Using Spiking Neural Networks [file 41537_2023_335_MOESM1_ESM.doc]

**Investigation of Social and Cognitive Predictors in Non-Transition Ultra-High-Risk’ Individuals for Psychosis Using Spiking Neural Network**

# Supplementary Information

1.1 Method:

In this study, the designed computational framework of Spiking Neural Network for predictive modelling on longitudinal social-cognitive data can be summarised in the following procedures:

1. Social--cognitive data interpolation.
2. Encoding the temporal data into sequences of spikes by
   1. Interpolating the data between points of measurements (forming time-series data).
   2. Encoding the time series data into spike trains.
3. Create an SNN 3D structure, defining and mapping the social-cognitive variable’s location.
4. Training the SNN model to learn the temporal information and forms pathways that can be interpreted as temporal patterns, which the SNN will use to classify/predict new information.
5. Training an output classifier to learn the relation between the SNN connectivity patterns and the cognitive data class labels (healthy control, remitted, converted, and maintained), and model visualisation.
6. Testing the SNN classifier on the individual $x$ data.
7. SNN model parameter optimisation.
8. Analysis of the connectivity of the trained SNN models for a better understanding of the patterns of interaction between social and cognitive variables over time, that define and predict an outcome.

The details of the designed SNN computational models including formulas and algorithms are presented in the following:

- ***Imputation and Interpolation of longitudinal Data***

To deal with missing values, we applied an imputation technique. With respect to the Euclidean distance metric, the K-nearest neighbour (KNN) method picked a subset of the most similar and comparable subjects to the one with missing values(1). The mean value of the distances was then assigned to the missing one. The original datasets were recorded at baseline, 6-months, 12-months, 18-months, and 24-months of follow-up, in the form of static behavioural data. To learn the changes in longitudinal data as a function of time in an SNN model, the data from each individual were linearly interpolated to time series by adding simulated data points that illustrate stepwise changes between every 2-time points, from T0 to T4. Here, 60-time points were interpolated for every 6-months period, representing ten data points per month (in total 245 data points were generated from each subject’s longitudinal data series). The obtained time points for the longitudinal data of an individual represent time series information which is used in this paper to create computational SNN models for predictive data modelling of social-cognitive outcomes. This interpolation is to transform the static data (measured at 5 points) into time series. The applied linear interpolation is based on a simple assumption to generate more data points between the original measurements while preserving the trend of data. There is no loss of trend-information in this method as the interpolated data points follow the trend of changes in the original data.

- ***Time Series Social-cognitive Encoding to Spikes***

To capture substantial upward and downward variations in the social-cognitive time series, the interpolated temporal patterns were encoded as sequences of binary events called spikes. The features values from each cognitive test are translated into a spike train (as demonstrated in Figure 1c). For a temporal cognitive test $C\left( t \right)$over time$t=1, 2,\ldots,n$, the variation of cognitive variable value over time is denoted by $B\left( t \right),$ where at baseline,$B\left( 1 \right)=C\left( 1 \right)$. At the next time point$t$, if the variable value is greater than $B\left( t-1 \right)$plus a threshold, $B\left( t-1 \right)+ \theta,$ then a positive spike is generated at $t$ and $B\left( t \right)$will be replaced by $B(t-1)$.

The encoding of positive and negative spikes is defined as follows:

| $\boldsymbol{spike}\left( \boldsymbol{t} \right)\mathbf{=}\left\{ \begin{aligned} \mathbf{1}\boldsymbol{then} \boldsymbol{B}\left( \boldsymbol{t} \right)\boldsymbol{\leftarrow}\boldsymbol{B}\left( \boldsymbol{t}\mathbf{-1} \right)\mathbf{+}\boldsymbol{\theta}\mathbf{;}\boldsymbol{if} \boldsymbol{C}\left( \boldsymbol{t} \right)\boldsymbol{\geq}\boldsymbol{B}\left( \boldsymbol{t}\mathbf{-1} \right)\mathbf{+}\boldsymbol{\theta} \\ \mathbf{-1}\boldsymbol{then B}\left( \boldsymbol{t} \right)\boldsymbol{\leftarrow}\boldsymbol{B}\left( \boldsymbol{t}\mathbf{-1} \right)\mathbf{-}\boldsymbol{\theta}\mathbf{;}\boldsymbol{if} \boldsymbol{C}\left( \boldsymbol{t} \right)\boldsymbol{\leq}\boldsymbol{B}\left( \boldsymbol{t}\mathbf{-1} \right)\mathbf{+}\boldsymbol{\theta} \\ \mathbf{0}\boldsymbol{otherwise} \end{aligned} \right.$ | (1) |
| --- | --- |

As shown in Relation (1), the encoded spike sequences are in the form of binary events, in which -1 refers to a negative spike (the site of downward changes in the cognitive variable values) and 1 is a positive spike (the site of upward changes). This method has been successfully used in dynamic vision sensors (DVS)(2). Figure 1c shows an example of encoding data into a sequence of positive and negative spikes using a threshold-based representation (TBR) algorithm. In this method, if the cognitive variable’s value change increases above a spike threshold at consecutive time moments, a positive spike is generated. On the other hand, if the cognitive variable’s values decrease below a threshold, then a negative spike is generated; otherwise, there is no spike generated. The generated spike trains represent changes in cognitive function that exceeded a threshold TBR^thr^.

- ***Initialisation of the SNN Model***

A 3-dimensional SNN model with 1000 neurons in created. For mapping the 23 cognitive variables in our dataset, 23 input neurons are randomly selected inside the SNN model. To map the data variables inro these 23 neurons, a Graph Matching technique (5) is used to compute the similarity between every pair of variables by measuring the correlation between their spike trains. Then, the more correlated variables are mapped into closer neurons in SNN model. After the mapping is completed, the SNN model is initialised with a Small-World Connectivity rule (SWC)(3). Based on the SWC rule, every neuron $i$ in the SNN model is probabilistically connected to other neurons that are located within a specific distance. The connection $w_{i,j}$ from neuron $n_{i}$ to $n_{j}$ is initially weighted as follows:

$w_{i,j}=\frac{r_{i,j}}{d_{i,j}}; r\in\mathbb{R}_{\left[ -1,1 \right]}$ (2)

where $d_{i,j}$ is the distance between two *neurons* $n_{i}$ and $n_{j}$.

The connection weights will later be adapted with respect to the incoming spikes through an unsupervised learning procedure. After a SNN model is initialised as above, two learning phases (unsupervised and supervised) are performed.

The SWC structure in which a probability of a neuron *i* to be connected to another neuron *j* inside the SNN depends on the distance between the two neurons, the larger the distance – the smaller the probability of connection. In some cases, a radius is defined which embodies the maximum distance of connections of one neuron to another in the 3D space of the SNN. The initial connections are assigned as small random weights so that, for example, 80% of them are weighted by positive values while 20% of them are weighted by negative values. These initial connection weights are then adjusted by unsupervised learning rules which rely on the temporal dynamics and spiking activity triggered by input nodes as explained in the next section(4).

- ***Unsupervised Learning in the 3D SNN Model***

Then, the unsupervised Spike-Time-Dependent Plasticity (STDP)(6) learning rule is used for learning in the SNN models. Through STDP learning, a connection $W_{i,j}$between node *i* and *j* are adapted according to the timing of their output spikes. If node *i* emits a spike earlier than *j*, then $W_{ij}$ will increase; otherwise, that would imply that node *j* is driving node *i* so $W_{ij}$ will decrease. STDP is a Hebbian learning that depends on the relative timing of pre- and postsynaptic action potentials. The STDP learning rule is defined using the following relation:

|  | $F\left( \Delta t \right)=\left\{ \begin{aligned} A_{+}exp(\Delta t/\tau_{+}) if \Delta t<0 \\ {-A}_{-}exp(-\Delta t/\tau_{-} ) if \Delta t\geq0 \end{aligned} \right.$ | (3) |
| --- | --- | --- |

where $F\left( \Delta t \right)$ defines the synaptic modification elicited from a single pair of pre- and postsynaptic spikes separated by a time interval $\Delta t=t_{pre}-t_{post}$. The parameters A_+_ and A_-_ define the maximum quantities of synaptic modification, which transpire when $\Delta t$ ≈ 0. The parameters $\tau_{+}$ and $\tau_{-}$determine the ranges of pre-to-post-synaptic inter spike intervals over which the synaptic strengthening and weakening occurs.

During this learning procedure, the input node will accumulate spikes to the SNN model and, if nodes cross an activation threshold, they will also emit output spikes. That spike is sent out to all the units it is connected with, and what reaches each distal node is the spike scaled by the connection weight. That node will likewise accumulate activity as a function of receiving spikes and, after crossing some threshold, fire. In such a way, spikes are transferred between nodes and propagated to the SNN model. Using this, the model captures the hidden spatiotemporal interactions between the cognitive variables over time, resulting in the identification of markers of UHR that are used to predict subsequent cognitive outcomes a few years ahead.

To compare two different trained SNN models (denoted by$S_{1}$and$S_{2}$) where each of them has $n$number of nodes in the model of SNN, the connection weight vectors (W) of S1 and S2, as two different trained SNN models, are subtracted as shown in the below formula.

$\boldsymbol{W}$(D) $=\boldsymbol{W}$(S1) $-\boldsymbol{W}$(S2) (4)

where $\boldsymbol{W}$(S1) is the connection weight vector of the model of the SNN model S1 and $\boldsymbol{W}$(S2) is the connection weight vector of the model of the SNN model S2. Here, we subtracted the SNN trained model of control group from remitters, converters and maintained.

- ***Supervised Learning and Classification using an SNN Classifier***

At this step, a dynamic evolving SNN (deSNN)(7) fully connected to all nodes in the 3D SNN, is used for classification of the activated spiking patterns in the 3D SNN when input longitudinal data are propagated through it. The deSNN applies supervised learning in an output classifier layer using the class labels of the training samples. For each sample in the training set, one node is evolved in the output layer and linked to all the nodes in the already trained 3D SNN. The connection $W_{ij}$ between node *i* from the 3D SNN and node *j* from the output layer is initialised by using a Rank-Order (RO)(8) rule. The RO rule emphasises a higher priority for earlier spikes to an output node. Data with class labels are propagated through the trained 3D SNN and a supervised learning process is applied to train an output classifier.

- ***SNN Models Parameter Optimisation***

For model parameter optimisation, an exhaustive grid search method has been utilised to minimise the cross-validation classification error. Each parameter will be searched within a range, specified by the minimum and maximum, through several iterations related to the number of steps for moving from minimum to maximum. For every model created out of N models, three main parameters (STDP learning rate, firing threshold, and classifier parameter mod) are chosen for optimisation. The parameters are selected by assigning 10 steps between the minimum and maximum values of each parameter. Therefore, for every model created, 1000 iterations of training (using N samples) and testing (using the single holdout sample) will be performed using a different combination of these three parameters. Then the parameters that result in the highest accuracy across the majority of iterations are reported as the optimal parameters. When the optimisation procedure is completed, the most selected values for the parameters across all the N models are selected as STDP learning rate= 0.01; firing threshold= 0.5; deSNN classifier parameter mod= 0.4.

**1.2 Data Variables**

The clinical and five neuropsychological cognitive tests batteries used in this study are defined as the following:

1. **The Comprehensive Assessment of At-Risk Mental States (CAARMS):** is a semi-structured assessment tool used by mental health professionals and researchers to identify help-seeking young people who are at ultra-high risk (UHR) of developing psychosis. (Cronbach’s α = .85).
2. **The Brief Assessment of Cognition in Schizophrenia (BACS):** The domains of cognitive function assessed by the BACS are those found to be consistently impaired and related to outcome in schizophrenia, that is, verbal memory, working memory, motor speed, verbal fluency (fruits, animal, and vegetables), attention, and executive function as designated by the MATRICS (Measurement and Treatment Research to Improve Cognition in Schizophrenia (MATRICS). (Cronbach’s α = .77)
3. **High-Risk Social Challenge (HiSoC):** The items on the HiSoC comprises three factors “Social-Interpersonal”, “Affect”, and “Odd Behaviour and Language” that are based on the original factor analysis of the task. The (Cronbach’s α = .89, .91, and .85 respectively).
4. **Perceptual Closure (PC):** The coherent perception of an object under circumstances when the visual information is incomplete. More recently the term has been expanded such that Drever's definition of closure refers to an all-embracing function "by means of which precepts, memories, actions attain stability." (Cronbach’s α = .80).
5. **Snakes in the Grass (SNK):** The snake detection theory (sometimes more correctly referred to as snake detection hypothesis) suggests that snakes have contributed to the evolution of primates' visual system. Snake stimuli are particularly distracting during perceptual tasks, suggesting that the brain preferentially processes snake stimuli, even when attentional processes are demanded by other targets. Snake enhanced detection was found also in young children. For psychosis (larger reaction time to snakes representing unconscious responses to snakes). (Cronbach’s α = .91)
6. **Continuous Performance Test (CPT):** Measures a person's sustained and selective attention. Sustained attention is the ability to maintain a consistent focus on some continuous activity or stimuli and is associated with impulsivity. (Cronbach’s α = .72).

# Supplementary Figures:


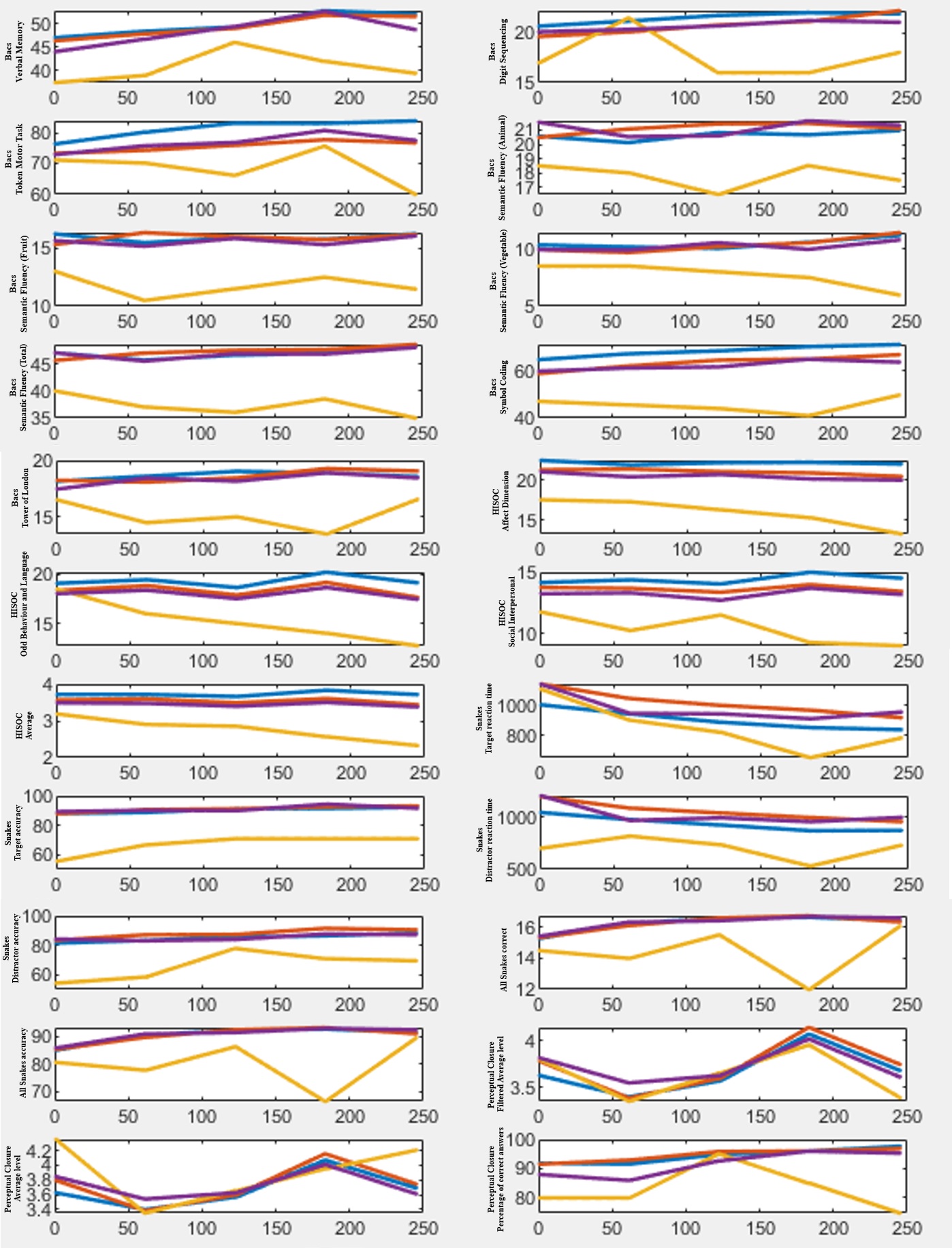


Figure 1. Longitudinal 23 cognitive data interpolation to time series across four groups: healthy (blue line), remitters (red line), maintained (purple line) and converters (yellow line, this is excluded from the experiments due to small number of samples). The left vertical axis is the mean values of each cognitive features across all individuals and horizontal axis is the timepoints of data collection (between 0-50 refer to baseline or T0; between 50-100 refer to T1 or after 6-months; between 100-150 refer to T2 or after 12-months; between 150-200 refer to T3 or after 18-months; and between 200-250 refer to T4 or after 24-months of data collection). This interpolation represents the trends of changes of the cogntive variables over time, so that patterns of these chnages can be learned in a SNN after encoding the time series into spike sequences.

Healthy Control Remitted Maintained


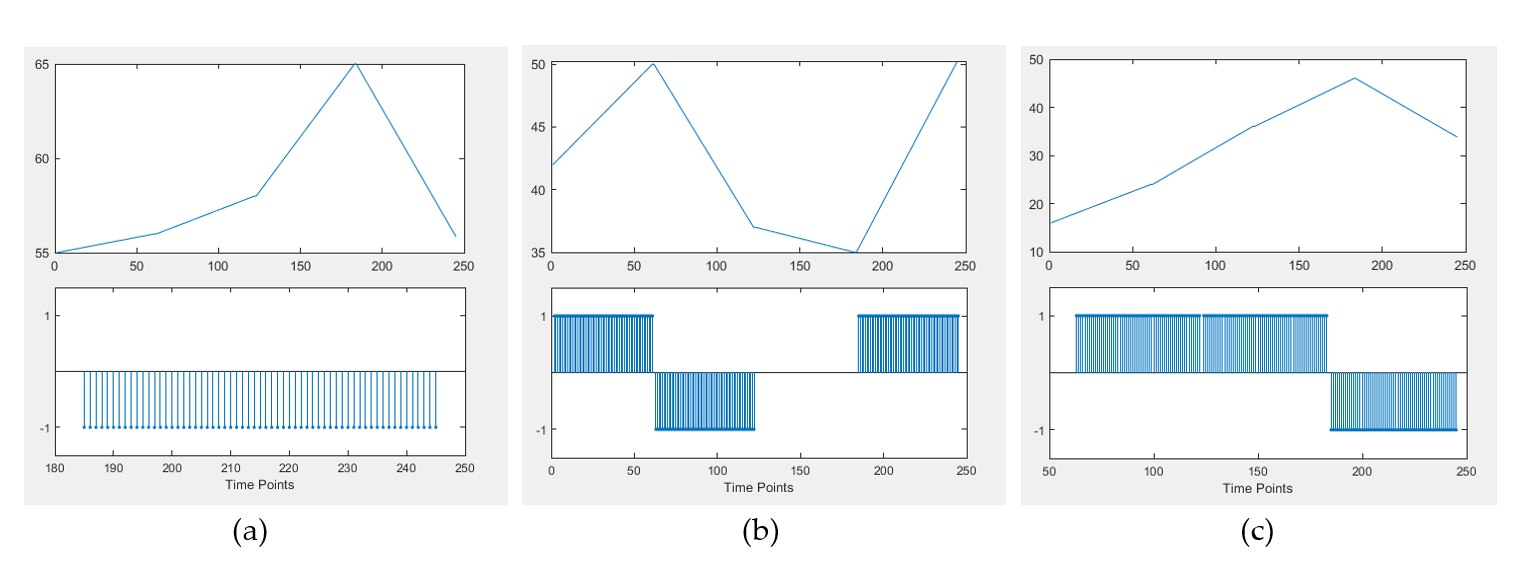


Encoded spikes

Interpolated Data

Figure 2. Examples of encoding interpolated time series from verbal memory feature to spike trains for group (a) (healthy control), group (b) (remitted) and group (c) (maintained) using the threshold-based-representation encoding method. This can be seen that certain changes (increases and decrease) in the time series are shown by spikes if the changes are greater than a threshold. These spikes are in a proper format to be used as inputs for SNN architecture. The encoding method here preserves the trend of significant changes (above a threshold) in the time series.

# References:

1. Beretta L, Santaniello A. Nearest neighbor imputation algorithms: a critical evaluation. BMC medical informatics and decision making. 2016;16(3):197-208.

2. Song S, Miller KD, Abbott LF. Competitive Hebbian learning through spike-timing-dependent synaptic plasticity. Nature neuroscience. 2000;3(9):919.

3. Liao X, Vasilakos AV, He YJN, Reviews B. Small-world human brain networks: perspectives and challenges. 2017;77:286-300.

4. Kasabov NKJNN. NeuCube: A spiking neural network architecture for mapping, learning and understanding of spatio-temporal brain data. 2014;52:62-76.

5. Gao Y, Tang J, Hong R, Yan S, Dai Q, Zhang N, et al. Camera constraint-free view-based 3-D object retrieval. 2011;21(4):2269-81.

6. Masquelier T, Guyonneau R, Thorpe SJJNc. Competitive STDP-based spike pattern learning. 2009;21(5):1259-76.

7. Kasabov N, Dhoble K, Nuntalid N, Indiveri GJNN. Dynamic evolving spiking neural networks for on-line spatio-and spectro-temporal pattern recognition. 2013;41:188-201.

8. Thorpe S, Gautrais J. Rank order coding. Computational neuroscience: Springer; 1998. p. 113-8.
